# Occurrence of a novel cleavage site for cathepsin G adjacent to the polybasic sequence within the proteolytically sensitive activation loop of the SARS-CoV-2 Omicron variant: The amino acid substitution N679K and P681H of the spike protein

**DOI:** 10.1371/journal.pone.0264723

**Published:** 2022-04-18

**Authors:** Zhadyra Mustafa, Hubert Kalbacher, Timo Burster

**Affiliations:** 1 Department of Biology, School of Sciences and Humanities, Nazarbayev University, Nur-Sultan, Kazakhstan Republic; 2 Institute of Clinical Anatomy and Cell Analysis, Faculty of Medicine, Eberhard Karls University of Tübingen, Tübingen, Germany; University of Central Florida, UNITED STATES

## Abstract

The serine proteases neutrophil elastase (NE), proteinase 3 (PR3), cathepsin G (CatG), and neutrophil serine protease 4 (NSP4) are secreted by activated neutrophils as a part of the innate immune response against invading pathogens. However, these serine proteases might be adopted by viruses to mediate viral surface protein priming resulting in host cell entrance and productive infection. Indeed, NE and PR3 hydrolyze the scissile peptide bond within the proteolytically sensitive polybasic sequence of the activation loop of SARS-CoV-2 located at the S1/S2 interface of the Spike (S) protein; an amino acid motif which differs from SARS-CoV-1. The occurrence of novel SARS-CoV-2 variants and substitution of distinct amino acids at the polybasic sequence prompts serious concerns regarding increased transmissibility. We propose that a novel cleavage site by CatG of the Omicron variant and the increased substrate turnover of the Delta variant by furin within the polybasic sequence should be considered for increased transmission of SARS-CoV-2 variants.

## Introduction

The severe acute respiratory syndrome coronavirus 2 Wuhan, WIV04 (SARS-CoV-2 Wuhan) and their variants, the causative agent of coronavirus disease 2019 (COVID-19), continuously mutates, leading to a possible increased transmission of the virus [1, 2]. The attachment of SARS-CoV-2 to the host cell is central in viral infectivity, thereby the Spike (S) protein represents a crucial determinant for binding to the receptor human angiotensin-converting enzyme 2 (hACE2) [3]. During viral replication the S1/S2 interface of the S protein is partly cleaved (primed) largely by furin in the infected producer cell, and the released virion is further activated by hydrolysis of S2’ subunit mainly by cell surface transmembrane protease serine subtype 2 (TMPRSS2) in order to generate the fusion peptide, which facilitates membrane fusion and lastly entrance of SARS-CoV-2 [4, 5]. Protease-mediated entry characterizes one of the main factors of successful productive infection of SARS-associated coronaviruses [6]. As a result, acquisition of novel occurring amino acid substitutions within the S protein might provoke an increased priming of SARS-CoV-2 and supports viral transmission. The prevalence of new SARS-CoV-2 variants compared to SARS-CoV-2 (Wuhan) might be explained by an increased proteolytic priming of the S protein at the polybasic region (preprint, https://doi.org/10.1101/2021.08.12.456173); amino acid substitutions at this site can be considered as an additional immune evasion strategy by SARS-CoV-2. A SARS-CoV-2 variant, referred to as SARS-CoV-2 (Alpha, B.1.1.7), comprises three deletions and seven substitutions in the S protein compared to the SARS-CoV-2 (Wuhan) [7]. SARS-CoV-2 (Kappa, B.1.617.1) as well as SARS-CoV-2 (Delta, B.1.617.2) harbor three key mutations in the S protein [8] and show a notable increase in transmissibility [9]. For instance, the Alpha variant augments transmissibility to 40–70% compared to the ancestral SARS-CoV-2 (Wuhan) virus, whereas the Delta variant appears to be 60% more transmissible than the Alpha variant [10]. Both Alpha and Delta variants have a mutation in the polybasic sequence, precisely at P681H and P681R, respectively [11] compared to the B.1.1.529 lineage SARS-CoV-2 (Omicron) which has two amino acid substitutions at the polybasic sequence: N679K and P681H. These substitutions have also been described for C.1.2 (N679K) and in 11% of cases of C.1.2 (N679K and P681H) (doi.org/10.1101/2021.08.20.21262342).

The fact that lymphocytopenia is observed with an increased prevalence in patients with a severe course of COVID-19 suggests that those inflammatory conditions, associated with mortality and morbidity, are caused by leukocytes rather than lymphocytes [12]. An increase in neutrophil counts and an elevated neutrophil-to-lymphocyte ratio (NLR) were postulated to be predictive for the prognosis of severe cases of COVID-19 [13]. Neutrophils, which infiltrate the site of infection creating innate immunity, are activated to release neutrophil serine proteases (NSPs). Of these, neutrophil elastase (NE), proteinase 3 (PR3), cathepsin G (CatG), and neutrophil serine protease 4 (NSP4) are resident in granules, and are recruited to the cell surface to be secreted to the extracellular space, which mediate host defense against various pathogens [14, 15]. Crucially, proteomic analysis of nasopharyngeal swabs of patients with SARS-CoV-2 revealed an increased amount of NE and CatG compared to non-infected individuals [16]. Additionally, neutrophil-derived NE and PR3 cleave the SARS-CoV-2 S protein adjacent to the polybasic insert, an amino acid motif encompassing RRAR, which suggests a role of S protein priming by NE and PR3 [17]. Thus, the frequency of NSPs in the nasal mucosa might contribute to S protein cleavage.

Here, we focused on the polybasic sequence of the proteolytically sensitive activation loop and examined whether the proteolytic cleavage by NSPs and furin is dependent on amino acid substation found in different SARS-CoV-2 variants.

## Material and methods

### *In silico* analysis of peptides

FASTA format sequences of the S protein peptides spanning the S1/S2 interface of SARS-CoV-1 (UniProtKB: P0DTC2) _660_YHTVSLLRSTSQKS_673_, SARS-CoV-2 (Wuhan, WIV04, UniProtKB: P59594) _678_TNSPRRARSVASQS_691_, SARS-CoV-2 P681H (Alpha) _678_TNS**H**RRARSVASQS_691_, SARS-CoV-2 P681R (Delta) _678_TNS**R**RRARSVASQS_691_, SARS-CoV-2 (C.1.2) _674_YQTQT**K**SPRRARSVASQS_691_, and SARS-CoV-2 (Omicron) _674_YQTQT**K**S**H**RRARSVASQS_691_ were retrieved from UniProtKB (https://www.uniprot.org). The peptide sequences were assessed for potential cleavage sites using *in silico* analysis. The peptides were evaluated using the program ExPASy Peptide Cutter (https://web.expasy.org/peptide_cutter/) against human NE (EC 3.4.21.37). This software predicts the possible cleavage sites of the given peptide sequence by distinct proteases.

### Analysis of peptide digestion *in vitro*

SARS-CoV-1 _660_YHTVSLLRSTSQKS_673_, SARS-CoV-2 (Wuhan) _678_TNSPRRARSVASQS_691_, SARS-CoV-2 P681H (Alpha) _678_TNS**H**RRARSVASQS_691_, SARS-CoV-2 P681R (Delta) _678_TNS**R**RRARSVASQS_691_, SARS-CoV-2 (Wuhan) _674_YQTQTNSPRRARSVASQS_691_, SARS-CoV-2 (Alpha) _674_YQTQTNS**H**RRARSVASQS_691_, SARS-CoV-2 (C.1.2) _674_YQTQT**K**SPRRARSVASQS_691_, or SARS-CoV-2 (Omicron) _674_YQTQT**K**S**H**RRARSVASQS_691_ peptides, spanning the polybasic sequence, were synthesized by EMC Microcollections GmbH (Tübingen, Germany) and purified by reversed-phase HPLC using a C18 250 x 8 column (Dr. Maisch GmbH, Ammerbuch-Entringen, Germany). The precise mass was determined by matrix-assisted laser desorption/ionization-time of flight (MALDI-TOF) mass spectrometry (Reflex IV, Bruker Daltonics, Bremen, Germany) with 2,5-Dihydroxybenzoic acid (DHB) matrix (Reflex IV, Bruker Daltonics). The indicated peptides were lyophilized and dissolved in PBS pH 7.4 (10 mg/ml).

200 μg/ml SARS-CoV peptides were incubated with human NE (4 μg/ml, Cat. No.: SE563, elastase from leukocytes of human sputum, Elastin Products Company Inc., EPC, Owensville, MO, USA or neutrophil-derived human NE, PN: 16-14-051200, Lot No. EH 2020–03, Athens Research and Technology, Athens, GA, USA), human CatG (4 μg/ml, Cat. No.: E-002, neutrophil-derived human CatG, BioCentrum Ltd., Krakow, Poland or neutrophil-derived CatG, PN: 16-14-030107, Lot No. CG 2017–01, Athens Research and Technology, Athens, GA, USA), CatG inhibitor (10 μM of Suc-Val-Pro-Phe^P^(OPh)_2_ (SucVPF) [18], 4 μg/ml (4 μl recombinant furin harboring 5 mM CaCl_2_ was added to 94 μl PBS pH 7.4 with 2 μl peptide) recombinant human furin (Cat. No. 450–47, Lot No. 1011516, Peprotech, Cranbury, NJ, USA), or 4 μg/ml PR3 (Athens Research and Technology, Athens, GA, USA) in PBS pH 7.4 for 2 h at 37°C. The resulting fragments from the digest were resolved by using an Intelligent Pump L-6200 (Merck-Hitachi, Darmstadt, Germany) connected to a Reprosil 100, 250 x 2 mm, C18 with 5 μm particle diameter column (Dr. Maisch GmbH, Ammerbuch-Entringen, Germany). The separation of the fragments from the peptide cleavage was performed by using a linear acetonitrile gradient and peptides were detected at 214 nm by UV vis detector L-4200 (Merck-Hitachi, Darmstadt, Germany) as well as a Chromato-Integrator D-2500 (Merck-Hitachi, Darmstadt, Germany).

The samples were analyzed by mass spectrometry (MALDI-TOF, Reflex IV, Bruker Daltonics, Bremen, Germany) and manually linked to predicted peptides created by an ExPASy FindPept tool (https://web.expasy.org/findpept/, Swiss Institute of Bioinformatics, Lausanne, Switzerland).

### Statistical analysis

One-way ANOVA for multiple comparison was used. Tukey HSD test for statistical significance was expressed as P-value < 0.05 equals * and P-value < 0.01 equals ** and is drawn in the diagram (VassarStats: Website for Statistical Computation, http://vassarstats.net).

## Results

**Fig 1:**
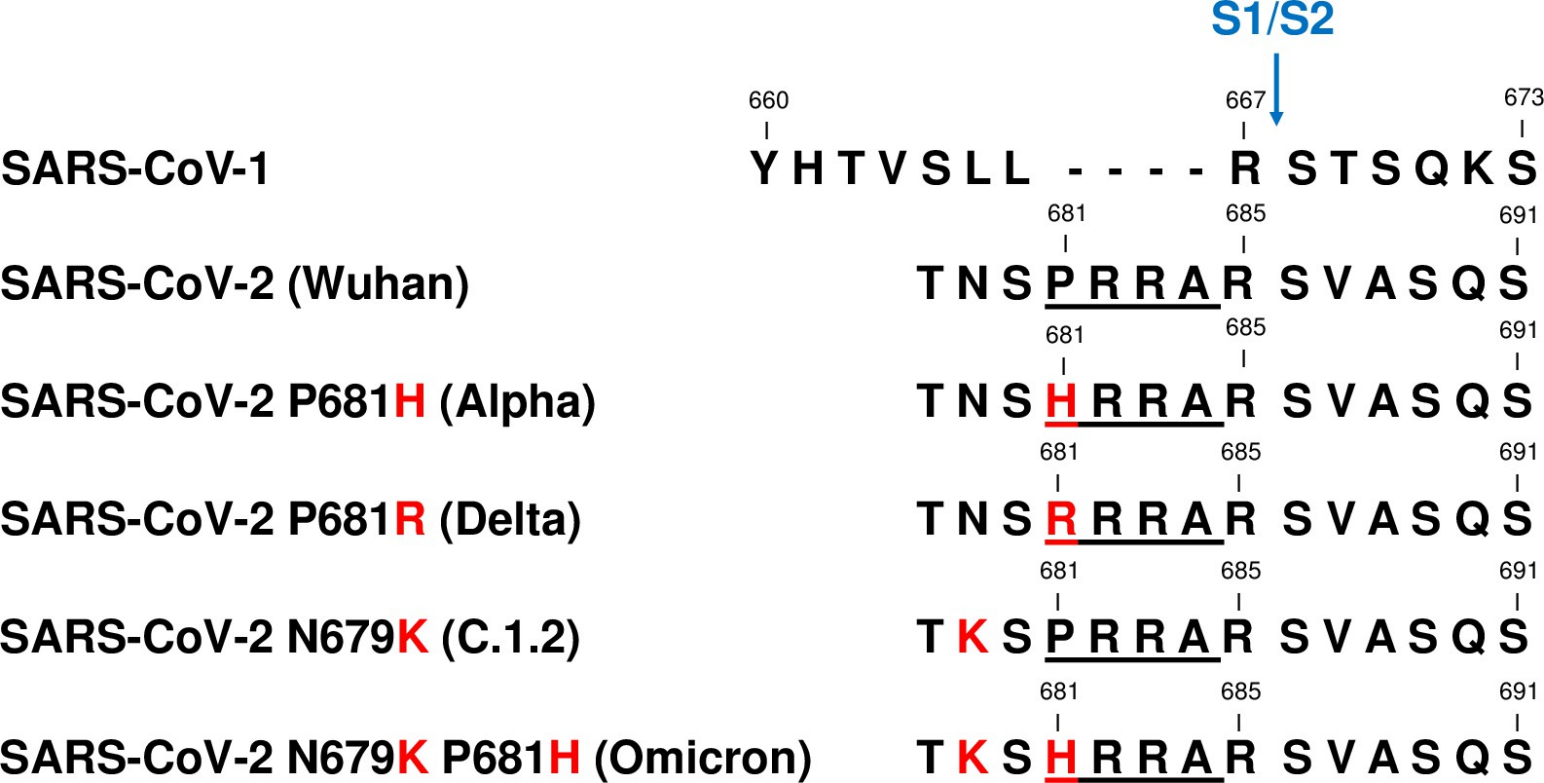
Amino acid alignment of the S1/S2 region of SARS-CoV-1 and SARS-CoV-2 (Wuhan, Alpha, Delta, C.1.2, and Omicron variants). The region corresponding to the S1/S2 cleavage site (blue arrow), four amino acid insert (underlined) and amino acid substitutions of SARS-CoV-2 variants (red) are shown. Accession numbers of sequences are mentioned in the material and methods section.

Protease-mediated SARS-CoV-2 entrance of the host cell is one of the major factors of a successful infection. The appearance of novel amino acid substitutions within the S protein might enhance viral transmission, escape natural- as well as vaccine-mediated immunity, and increase pathogenicity [19, 20]. Histamine or arginine substitutions might generate further cleavage sites within the proteolytically sensitive activation loop (Fig 1).

**Fig 2:**
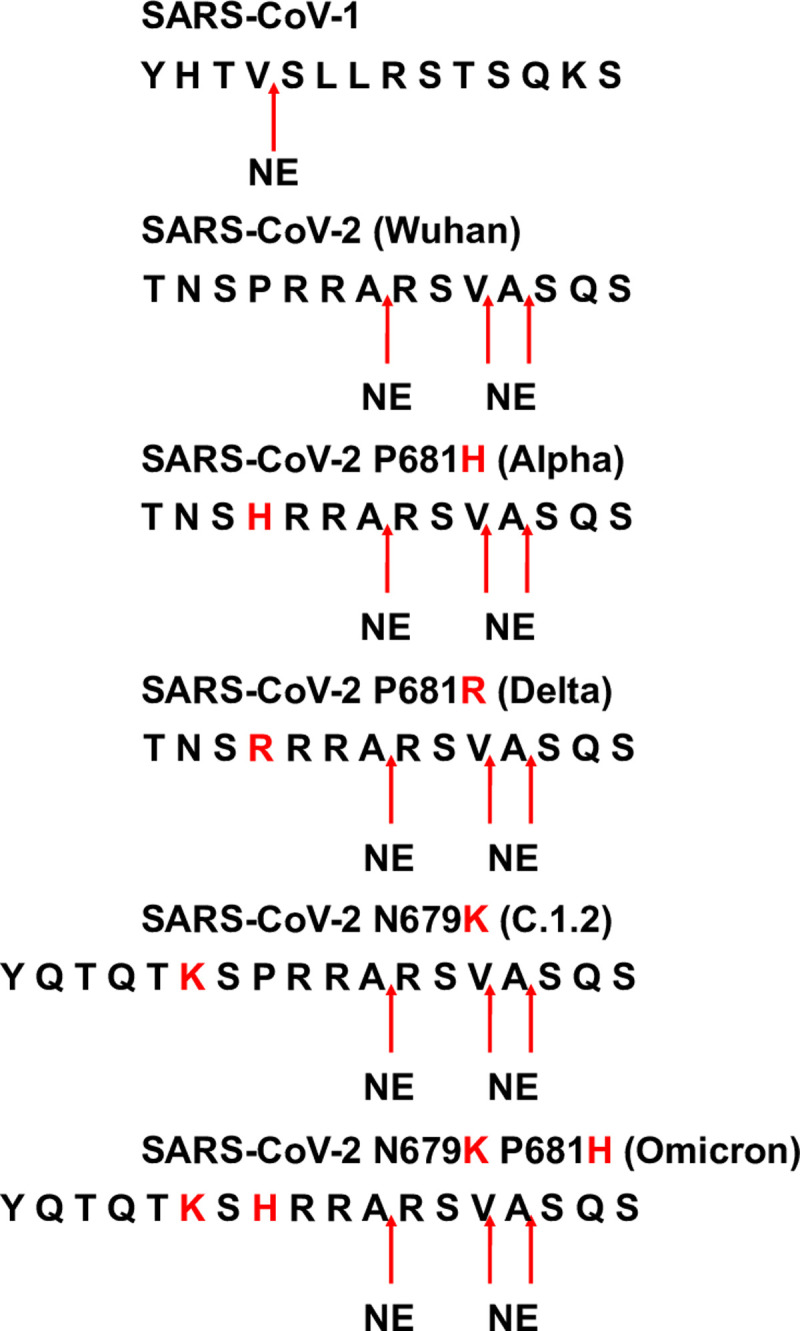
NE cleavage site prediction covering the polybasic sequence of the activation loop of the S protein. Potential cleavage sites of NE at the proteolytically sensitive activation loop of SARS-CoV-1, SARS-CoV-2 (Wuhan), SARS-CoV-2 P681H (Alpha), SARS-CoV-2 P681R (Delta), SARS-CoV-2 N679K (C.1.2), and SARS-CoV-2 N679K P681R (Omicron) peptides were mapped using “ExPASy peptide cutter”. Red arrows indicate cleavage sites.

In a first set of investigations, a prediction approach of cleavage sites for the SARS-CoV-1 _660_YHTVSLLRSTSQKS_673_, SARS-CoV-2 (Wuhan) _678_TNSPRRARSVASQS_691_, SARS-CoV-2 P681H (Alpha) _678_TNS**H**RRARSVASQS_691_, and SARS-CoV-2 P681R (Delta) _678_TNS**R**RRARSVASQS_691_, SARS-CoV-2 N679K (C.1.2) _674_YQTQT**K**SPRRARSVASQS_691_, and SARS-CoV-2 N679K P681R (Omicron) _674_YQTQT**K**S**H**RRARSVASQS_691_. peptides, spanning the polybasic sequence of the S1/S2 interface, were performed for NE by using ExPASy (https://web.expasy.org/peptide_cutter/). Based on these outcomes, NE processed the SARS-CoV-1 peptide at _663_VS_664_ and the variants of SARS-CoV-2 peptides between _684_AR_685_, _687_VA_688_, and _688_AS_689_ (Fig 2). Of note, only NE for substrate cleavage prediction was available in ExPASy for NSPs.

**Fig 3:**
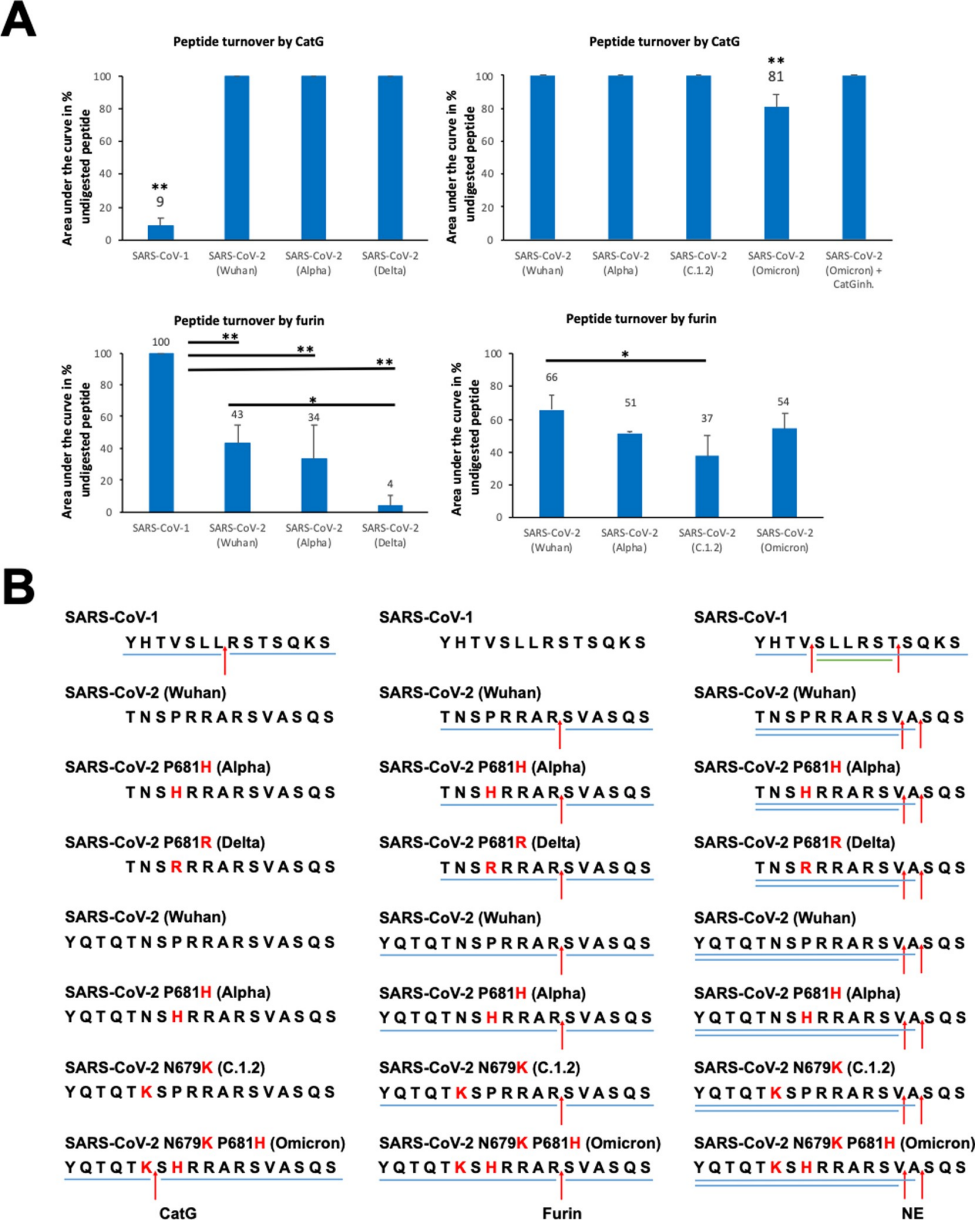
Proteolytic cleavage sites within the polybasic sequence of the proteolytically sensitive activation loop. **A)** The peptides (200 μg/ml) were incubated with 4 μg/ml of the respective protease for 2h at 37°C and followed by quantification of the peptide turnover by using HPLC. Of note, quantification of substrate (SARS-CoV-2 variants) turnover by NE was not completely feasible due to the fact that the undigested peptide and the digestion pattern migrated on the same HPLC peak. The summary of substrate turnover by CatG (SARS-CoV-2 Omicron, n = 5) or furin (n = 3) is shown in a bar diagram. P < 0.05 (*) or P < 0.01 (**). CatG inhibitor = CatGinh. **B)** Catalytic cleavage sites of CatG, furin, and NE are summarized. Red arrows indicate cleavage sites and blue bars the digestion pattern (peptides) detected by mass spectrometry. Of note, green bars represent less prominent peptides. CatG, furin, and NE at least n = 2 independent experiments.

In order to verify these results experimentally, SARS-CoV-1, SARS-CoV-2 (Wuhan), SARS-CoV-2 P681H (Alpha), and SARS-CoV-2 P681R (Delta), SARS-CoV-2 N679K (C.1.2), and SARS-CoV-2 N679K P681R (Omicron) peptides were synthesized and incubated with the indicated proteases (Fig 3). The resulting fragments were resolved as well as quantified by HPLC (Fig 3A) and analyzed by using mass spectrometry (Fig 3B). CatG digested SARS-CoV-1 peptide, in contrast to the different SARS-CoV-2 peptides (Wuhan, Alpha, Delta, C.1.2) which failed to be hydrolyzed by CatG (Fig 3A, upper panel). However, CatG cleaved SARS-CoV-2 N679K P681R (Omicron) peptide with a substrate turnover rate of approximately 20%. The substrate turnover of SARS-CoV-2 (Wuhan), SARS-CoV-2 P681H (Alpha), and SARS-CoV-2 P681R (Delta) by furin was increased when compared to SARS-CoV-1; however, the turnover rate was only significant for SARS-CoV-2 P681R (Delta) and SARS-CoV-2 P681R (C.1.2) as compared to SARS-CoV-2 (Fig 3A, lower panel).

Next we sought to determine the precise position where these peptides were hydrolyzed. CatG cleaved the SARS-CoV-1 peptide bond between _666_LR_667_ [17] (Fig 3B, left panel; S1 Fig). Strikingly, a novel cleavage site was detected for SARS-CoV-2 N679K P681R (Omicron) peptide between _679_KS_680_. The serine protease furin did not digest SARS-CoV-1, in contrast to SARS-CoV-2 peptides which were hydrolyzed between _685_RS_686_ (Fig 3B, middle panel; S1 Fig). NE digested SARS-CoV-1 between _663_VS_664_ and were less prominent between _669_TS_670_ (Fig 3B, right panel; S1–S3 Figs). NE catalyzed the hydrolysis of all SARS-CoV-2 variants at _687_VA_688_ and _688_AS_689_. PR3 cleaved the peptides between _687_VA_688_ (S4 and S5 Figs).

**Fig 4:**
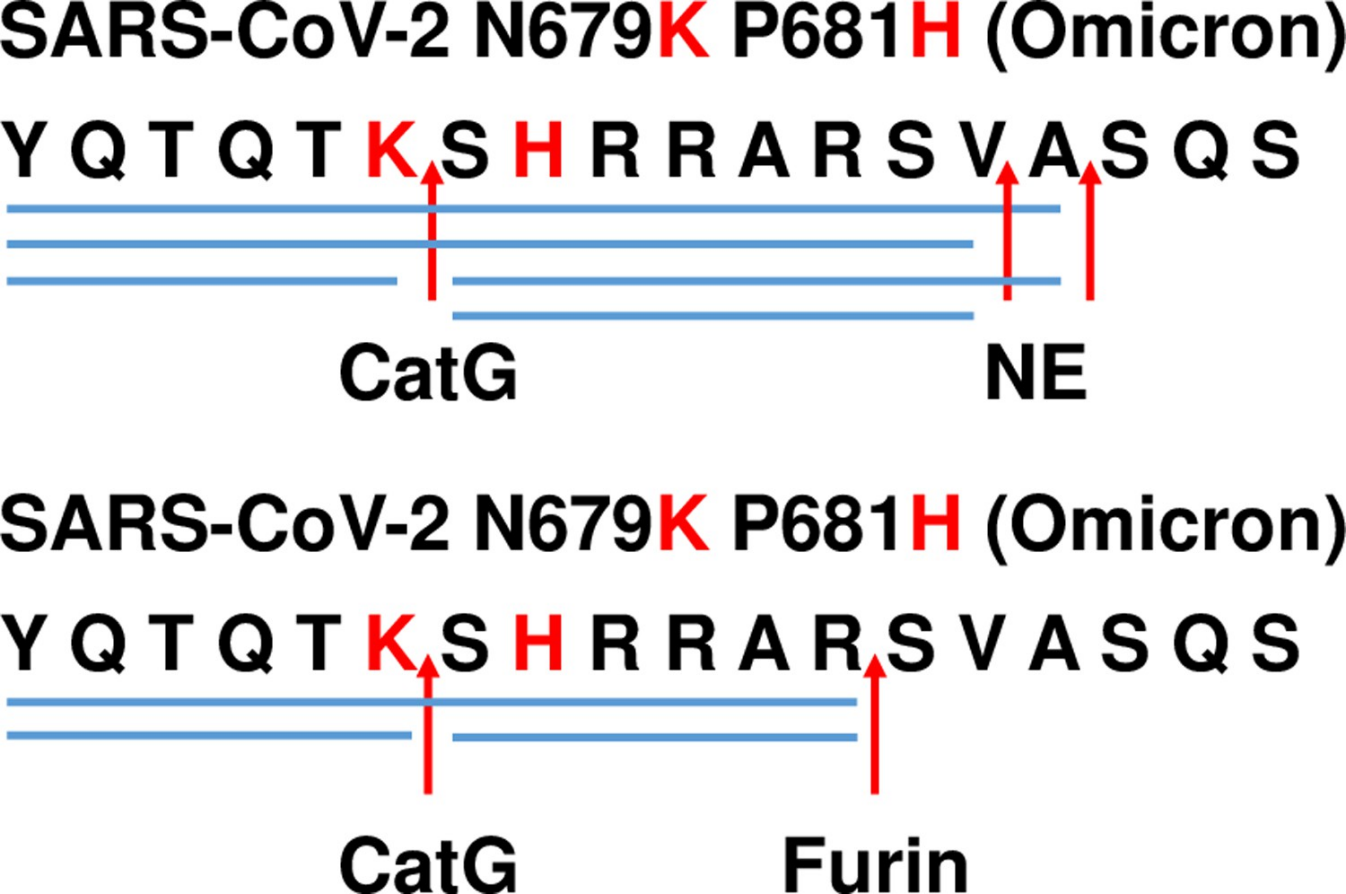
Proteolytic cleavage sites by the combined action of CatG, furin, and NE. 200 μg/ml SARS-CoV-2 N679K P681R (Omicron) peptide was incubated with equal amounts of CatG and NE (4 μg/ml, upper panel) or 4 μg/ml of CatG, furin, and NE for 2h at 37°C. The peptide turnover was determined by HPLC and mass spectrometry (n = 2).

In an additional experiment, we tested whether the digestion pattern might change when SARS-CoV-2 N679K P681R (Omicron) peptide will be incubated with CatG and NE or CatG, furin, and NE. Indeed, furin largely directed the processing of the peptide in contrast to NE, since NE generated fragments were not detected (Fig 4).

## Discussion

According to the ExPASy data, NE cleaves the polybasic sequence of the proteolytically sensitive activation loop of SARS-CoV-2 Wuhan, Alpha, and Delta variants at three different positions (Fig 2). NE hydrolyzed the SARS-CoV-1 peptide after threonine (T679) since NE has considerable preference for this amino acid at P1 [21]. This is a limitation of the prediction tool since only the P1 position of the peptide and a preference for valine and alanine is considered for NE, underlying the importance for an experimental verification of the obtained data.

The amino acid substitution increased the digestion capacity of furin (Wuhan → Alpha → Delta, Fig 3A) within the polybasic sequence which might be one reason for increased transmission. Whether the novel cleavage site performed by CatG for SARS-CoV-2 Omicron peptide, carrying the mutations N679K and P681H, might increase infectivity and transmissibility need to be investigated by a cell-based assay. An additional limitation of our investigation is the fact that the complete S protein instead of peptides should be used in antigen processing. Furthermore, the increased turnover capacity of furin and the fact that furin (CatG) controlled the processing of SARS-CoV-2 N679K P681R (Omicron) peptide in contrast to NE, indicate to target furin and, most likely, CatG with selective protease inhibitors as a logical consequence to interfere with the priming of the S protein.

One report suggested that TMPRSS2 is the protease responsible for the cleavage of the polybasic insert of SARS-CoV-2 [22]. Other studies have considered that furin digests at this site and TMPRSS2 at the S2’ position [4, 20]. However, knockout of furin in target cells did not substantially affect the cleavage of the S1/S2 interface, and loss of furin function reduced but not completely prevented SARS-CoV-2 infection of host cells, indicating that other cellular proteases additionally prime the S protein [23]; possibly NE and in the case of SARS-CoV-2 Omicron CatG.

Substrate recognition and specificity by proteases can be altered by glycans [24]. It has been suggested that proline at the position 681 in SARS-CoV-2 allows an addition of O-linked glycans to nearby residues, leading to the creation of a mucin-like domain that shields antigen processing [25], which is circumvented by the introduction of an arginine residue in SARS-CoV-2 P681R. Our data did not indicate an increased cleavage of the polybasic sequence of the Alpha variant by furin (Fig 3B) or NE (S3 Fig), the enhanced transmissibility might be rather true by elevated receptor-binding to the S protein [26], even though these findings are controversial [27], or additional proteases might be responsible for higher transmissibility of SARS-CoV-2 (Alpha). Strikingly, the P681R substitution is crucial for viral replication which has been suggested by reverting the P681R substitution to a wild-type P681 of the SARS-CoV-2 (Delta) background. Moreover, the higher replication of the Delta variant cannot be explained by the possibly enhanced S protein/ACE2 receptor binding since RBD of the Alpha variant has a higher affinity to ACE2 in contrast to the RBD of the Delta variant. The author hypothesized that furin might be responsible for increased transfection by enhanced priming of the S protein at the P681R substitution (preprint, https://doi.org/10.1101/2021.08.12.456173), which could be explained by our findings of increased SARS-CoV-2 P681R (Delta) substrate turnover by furin. Of note, the hydrolysis of the S1/S2 interface by furin, located in the trans-Golgi network, provokes a partial release of the S1 subunit of the S protein trimer [28].

## Conclusions

Previously, a study demonstrated that the infection rate of SARS-CoV-1 was increased by porcine pancreas-derived elastase [6], NE and CatG levels were increased in nasopharyngeal swabs of SARS-CoV-2 patients in comparison to the control group [16], and our *in vitro* data using NSPs and furin support the concept that proteolytic digestion of the S protein adjacent to the polybasic sequence play a role in priming of the S protein in an early event and might be one of several reasons for increased transmission of novel variants. Hypothetically, such priming by neutrophil-derived serine proteases and novel occurring amino acid substitution in the SARS-CoV-2 S protein can be understood as an immune evasion strategy by the virus.

## Supporting information

S1 Fig Mass spectrometry data. Summary of the mass spectrometry data (the masses of undigested peptides were not included). The digestion pattern was analyzed by HPLC and mass spectrometry.
(PPTX)

S2 Fig Processing of SARS-CoV-1 peptide by NE and PR3. The SARS-CoV-1 peptide was incubated with NE or PR3 and the digestion pattern was quantified by HPLC.
(PPTX)

S3 Fig Processing of SARS-CoV-2 Wuhan and SARS-CoV-2 Alpha peptides by NE. SARS-CoV-2 Wuhan and SARS-CoV-2 Alpha peptides were incubated with NE and the digestion pattern was quantified by HPLC.
(PPTX)

S4 Fig Processing of SARS-CoV-2 (Wuhan), SARS-CoV-2 P681H (Alpha), SARS-CoV-2 N679K (C.1.2), and SARS-CoV-2 N679K P681R (Omicron) peptides by PR3. Peptides were incubated with PR3 and the digestion pattern was analyzed by HPLC and mass spectrometry, (n = 2).
(PPTX)

S5 Fig Summary of statistical analysis. One-way ANOVA was used for the statistical analysis of the data found in Fig 3.
(PPTX)

## Abbreviations

Cat: cathepsin
CatG: cathepsin G
COVID-19: coronavirus disease 2019
NE: neutrophil elastase
NSPs: neutrophil serine proteases
NSP4: neutrophil serine protease 4
PR3: proteinase 3
SARS-CoV-2: severe acute respiratory syndrome coronavirus 2
S protein: spike protein
TMPRSS2: transmembrane protease serine subtype 2
