# Supplementary figures and images for: Occurrence of a novel cleavage site for cathepsin G adjacent to the polybasic sequence within the proteolytically sensitive activation loop of the SARS-CoV-2 Omicron variant: The amino acid substitution N679K and P681H of the spike protein
Source: PLoS One. 2022 Apr 18;17(4):e0264723. doi: 10.1371/journal.pone.0264723 (PMC9015119; doi:10.1371/journal.pone.0264723)

## Slide 1
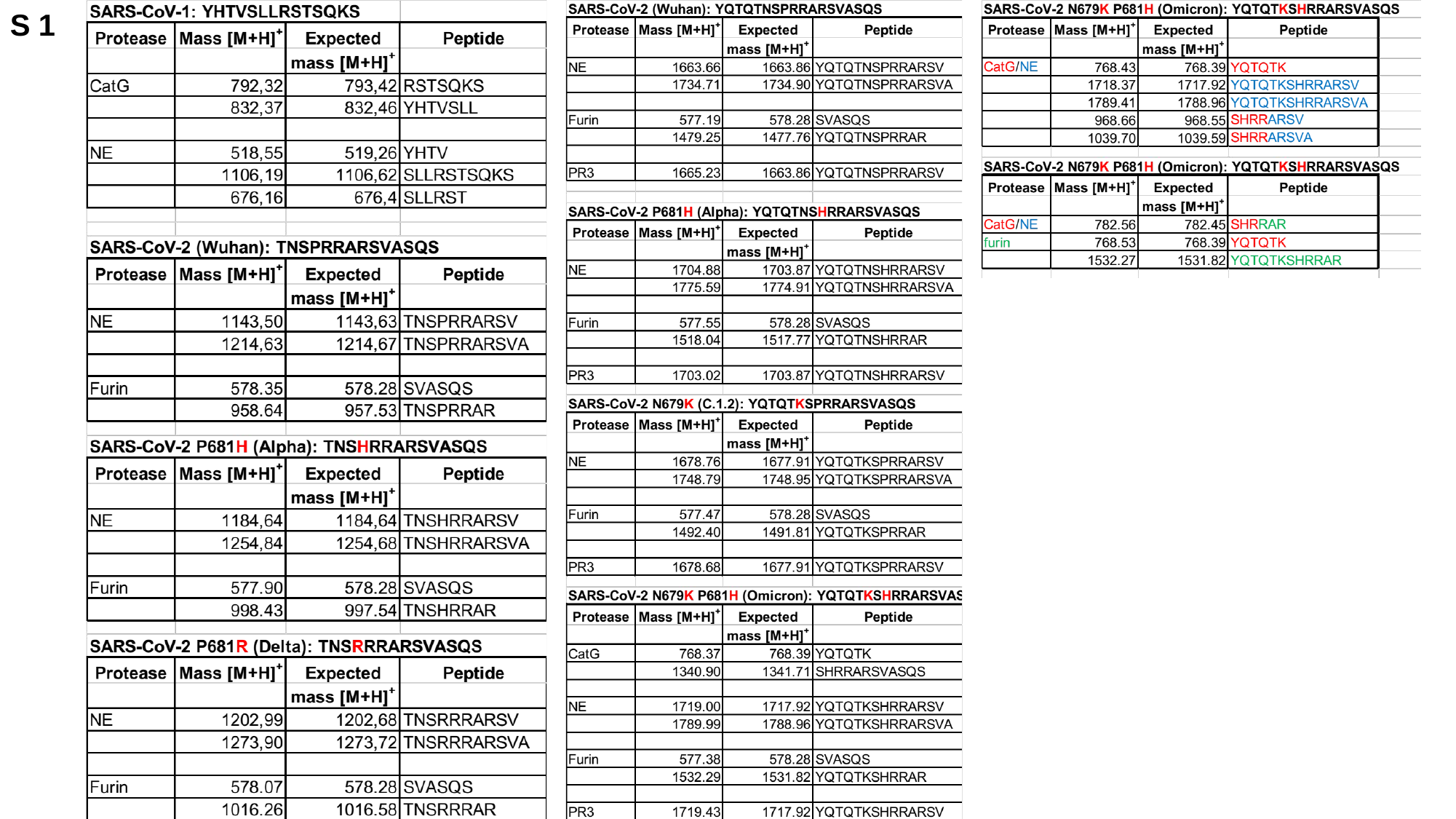

S 1

Supplement: S1 Fig — Summary of the mass spectrometry data (the masses of undigested peptides were not included). The digestion pattern was analyzed by HPLC and mass spectrometry. (PPTX) [file pone.0264723.s001.pptx]

## Slide 1
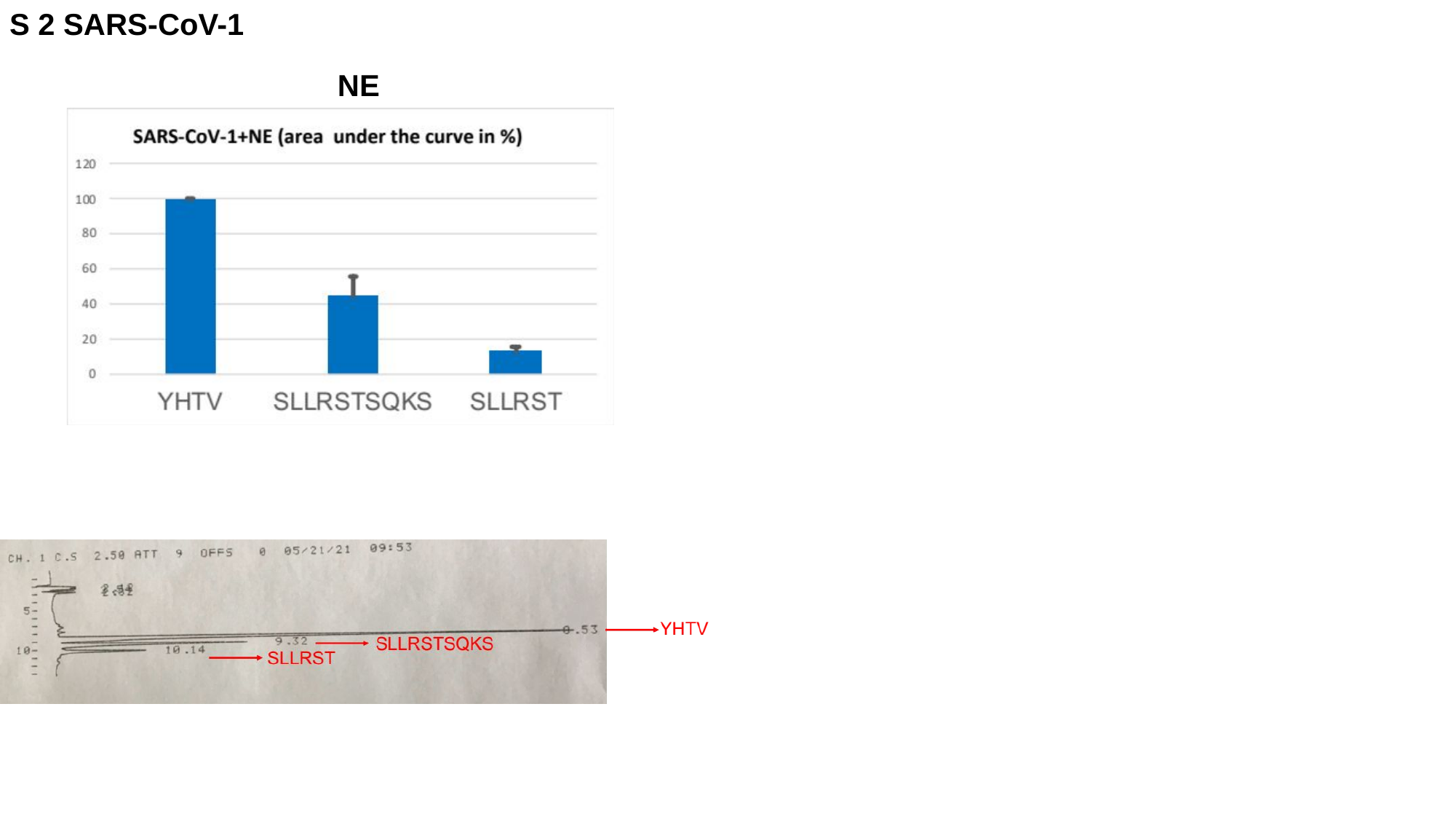

S 2 SARS-CoV-1
NE

Supplement: S2 Fig — The SARS-CoV-1 peptide was incubated with NE or PR3 and the digestion pattern was quantified by HPLC. (PPTX) [file pone.0264723.s002.pptx]

## Slide 1
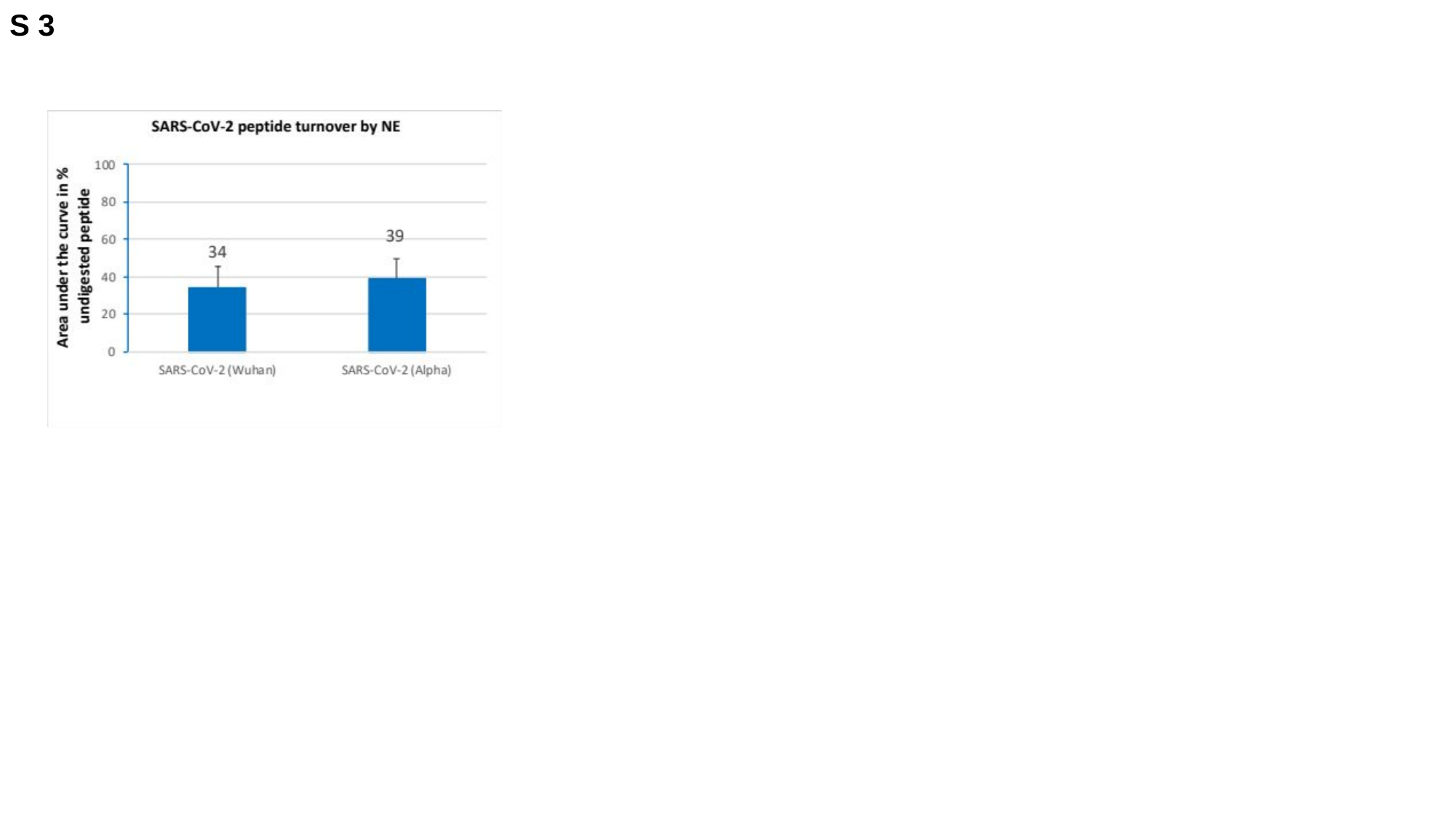

S 3

Supplement: S3 Fig — SARS-CoV-2 Wuhan and SARS-CoV-2 Alpha peptides were incubated with NE and the digestion pattern was quantified by HPLC. (PPTX) [file pone.0264723.s003.pptx]

## Slide 1
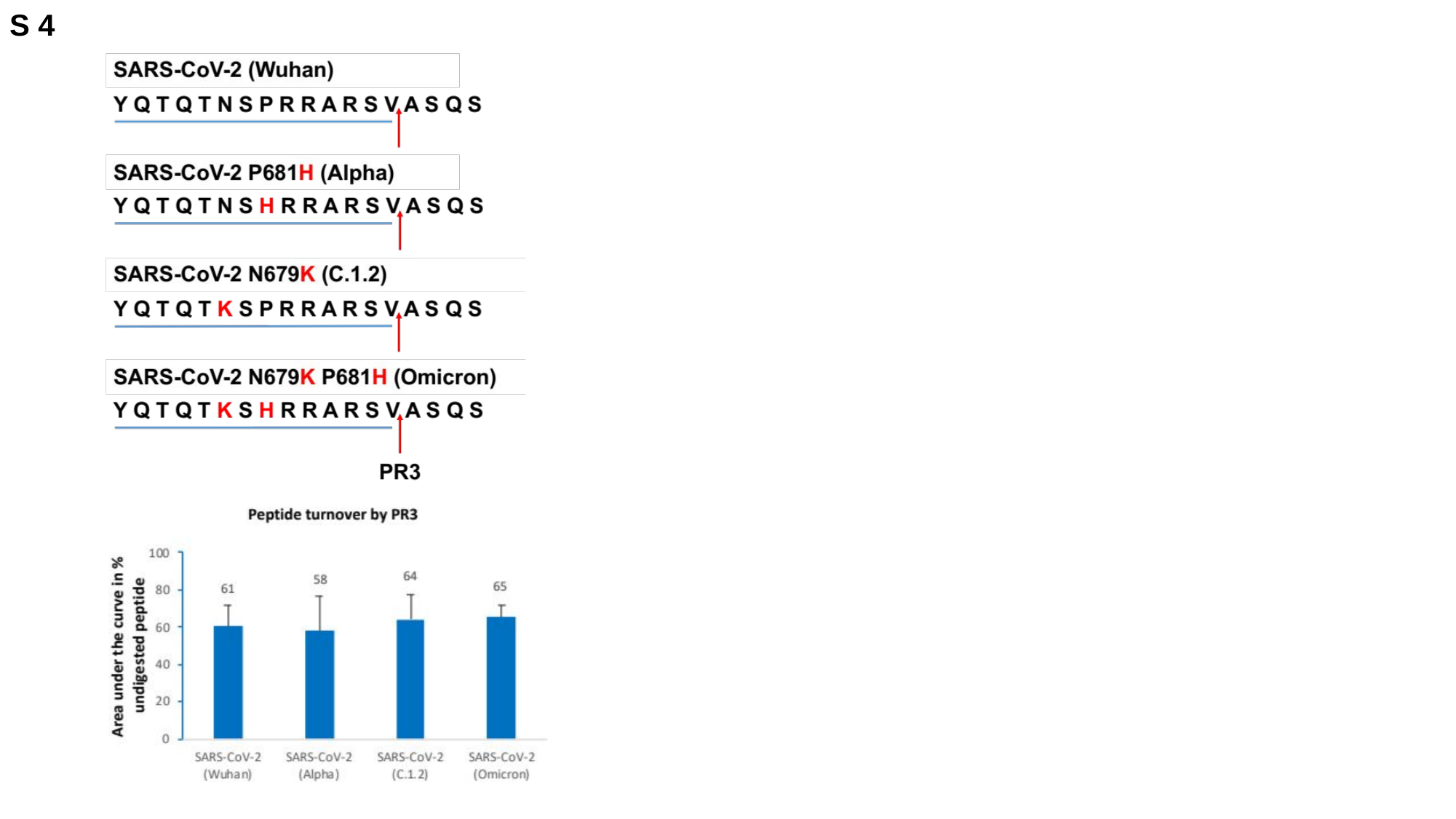

S 4

Supplement: S4 Fig — Peptides were incubated with PR3 and the digestion pattern was analyzed by HPLC and mass spectrometry, (n = 2). (PPTX) [file pone.0264723.s004.pptx]

## Slide 1
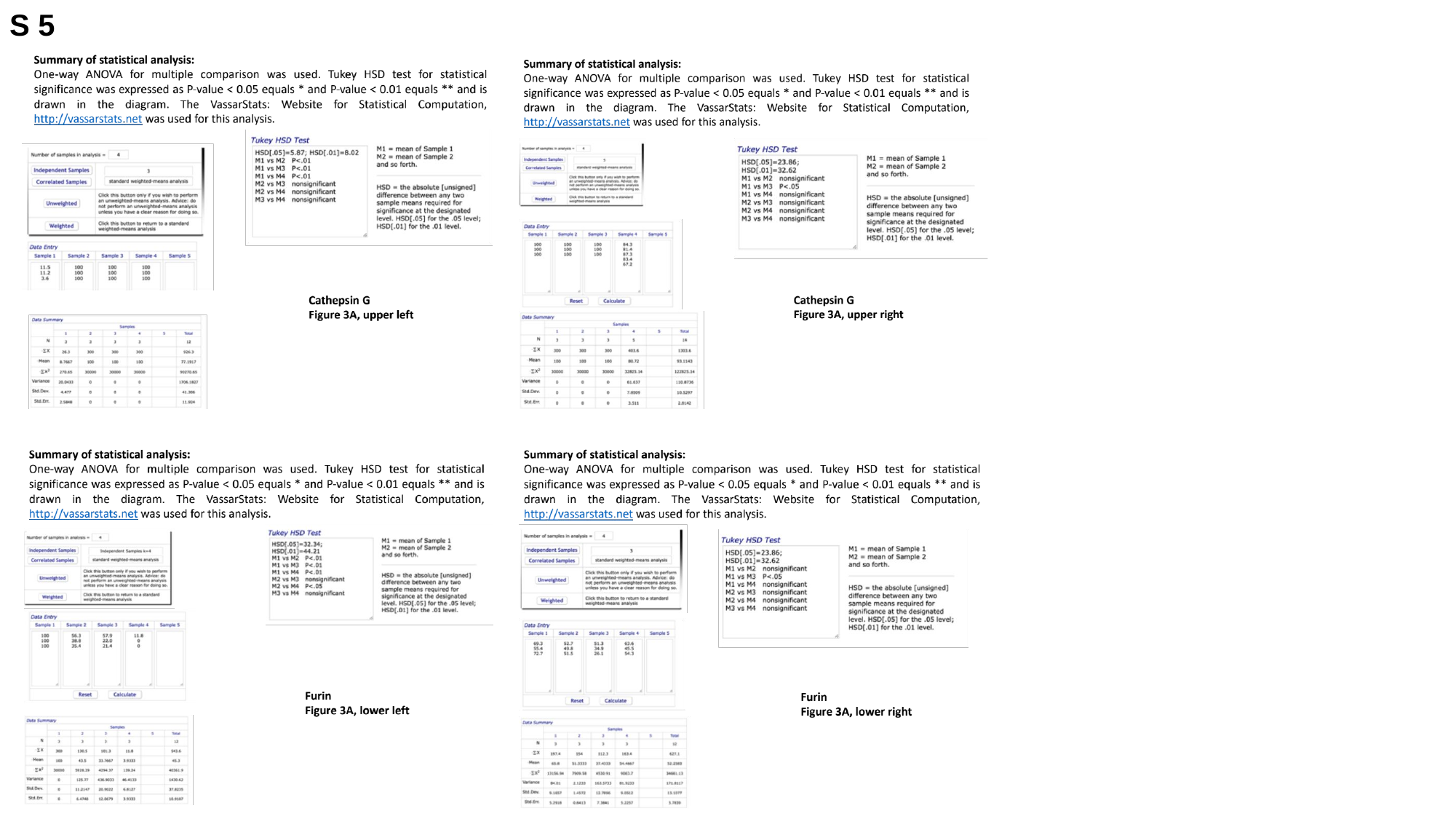

S 5

Supplement: S5 Fig — One-way ANOVA was used for the statistical analysis of the data found in Fig 3. (PPTX) [file pone.0264723.s005.pptx]
